# Mobile Apps in Oncology: A Survey on Health Care Professionals’ Attitude Toward Telemedicine, mHealth, and Oncological Apps

**DOI:** 10.2196/jmir.6399

**Published:** 2016-11-24

**Authors:** Kerstin A Kessel, Marco ME Vogel, Friederike Schmidt-Graf, Stephanie E Combs

**Affiliations:** ^1^ Klinikum rechts der Isar Department of Radiation Oncology Technical University of Munich (TUM) Munich Germany; ^2^ Institute for Innovative Radiotherapy (iRT) Department of Radiation Sciences (DRS) Helmholtz Zentrum München (HMGU) Neuherberg Germany; ^3^ Klinikum rechts der Isar Department of Neurology Technical University of Munich (TUM) Munich Germany

**Keywords:** mHealth, eHealth, telemedicine, mobile application, app, smartphone, oncology, patient-reported outcome

## Abstract

**Background:**

Mobile apps are an evolving trend in the medical field. To date, few apps in an oncological context exist.

**Objective:**

The aim was to analyze the attitude of health care professionals (HCPs) toward telemedicine, mHealth, and mobile apps in the field of oncology.

**Methods:**

We developed and conducted an online survey with 24 questions evaluating HCPs’ general attitude toward telemedicine and patients using medical mobile apps. Specific questions on the possible functionality for patients and the resulting advantages and disadvantages for both the patients’ and HCPs’ daily clinical routine were evaluated.

**Results:**

A total of 108 HCPs completed the survey. In all, 88.9% (96/108) considered telemedicine useful and 84.3% (91/108) supported the idea of an oncological app complementing classical treatment. Automatic reminders, timetables, and assessment of side effects and quality of life during therapy were rated as the most important functions. In contrast, uncertainty regarding medical responsibility and data privacy were reasons mostly named by critics. Most (64.8%, 70/108) were in favor of an alert function due to data input needing further clarification, and 94% (66/70) were willing to contact the patient after a critical alert. In all, 93.5% (101/108) supported the idea of using the collected data for scientific research. Moreover, 75.0% (81/108) believed establishing a mobile app could be beneficial for the providing hospital.

**Conclusions:**

A majority of HCPs are in favor of telemedicine and the use of oncological apps by patients. Assessing side effects can lead to quicker response and thus lower inconvenience for patients. Clinical data, such as life quality and treatment satisfaction, could be used to evaluate and improve the therapy workflow. Eventually, a mobile app would enhance the patients’ relationship to their treating department because they are in permanent contact.

## Introduction

For younger generations, it is impossible to imagine an everyday life without mobile phones. The estimated number of those devices will exceed 2.16 billion in 2016 [1]. In the last decade, apps for mobile phones and tablets have changed our life immensely. Currently, more than 2.2 million apps [2] are available in the Google Play store and approximately 1.8 million apps [3] are available in the Apple App Store. Both distribute nearly 70,000 apps each in the category Health and Fitness, and approximately 33,000 and 46,000 each, respectively, as medical apps [2,3]. Apps for chronic diseases, mental health, or fitness are forthcoming [4-6]. Gadgets to track blood sugar, heart rate, or body weight are used more commonly. For the medical field, the World Health Organization (WHO) defines these tools as mHealth or “medical and public health practice supported by mobile devices, such as mobile phones, patient monitoring devices, personal digital assistants, and other wireless devices” [7].

It is apparent the willingness to use mHealth apps or devices is high and the need is growing [8]. mHealth is always closely associated with telemedicine, which the WHO defines as: “The delivery of health care services, where distance is a critical factor, by all health care professionals using information and communication technologies for the exchange of valid information for diagnosis, treatment, and prevention of disease and injuries, research and evaluation...” [9].

Practicing mHealth as a patient-assisting approach only is not expedient. Rather, mHealth with professionally advised telemedical services as a holistic concept of diagnostics and treatment is the objective of further development.

Recently, Denis et al [10] showed a significant improvement in overall survival in patients with high-risk lung cancer using a mobile-friendly Web app. In a randomized controlled trial, they compared patients using an app for self-scoring symptoms to those in a nonintervention arm. Median overall survival was 19 months versus 12 months, respectively. It was discussed that due to the regular patient self-reported outcome, earlier medical care could be achieved. Prior publications by Denis et al [11] showed higher compliance, and even 5 weeks’ earlier detection of relapse, by using an Internet-based app.

To date, few native apps for mobile phones or tablets in an oncological context exist that support cancer management or cancer patients themselves during therapy as well as follow-up and allow for data analysis and/or direct feedback about therapy parameters [12,13]. A recent review by Brouard et al [13] identified 117 apps for patients, mostly for oncological information and treatment monitoring. The scientific validation (mentioned in the store description) of those apps was poor (27.4%, 32/117). Collado-Borrell et al [14] evaluated 166 apps (Android: n=75; Apple: n=59; both: n=32) for cancer patients. The purposes of the apps were mainly informative (39.8%, 66/166), diagnostic (38.6%, 64/166), and preventive (28.3%, 47/166). Moreover, the study showed a lack of involvement by qualified professionals, as only 48.8% (81/166) were developed by health care organizations. There is an ongoing discussion whether apps are really valuable and whether health care professionals (HCPs) will accept the use of them by patients in clinical day-to-day life. Therefore, we initiated a survey to evaluate the opinions of HCPs on oncological apps within our Oncology Center (Onkologisches Zentrum am RHCCC am MRI Technical University Munich, Munich, Germany). This paper analyzes the general attitude of HCPs toward mHealth, oncological apps, and their use by patients.

## Methods

A team of experienced oncologists and medical computer scientists developed a questionnaire containing 24 questions evaluating opinions on the use of mHealth and mobile apps in an oncological context at the Technical University Munich, Klinikum rechts der Isar. Focus was on HCPs’ general attitude toward telemedicine and patients using medical mobile apps using specific questions on functionality and the possible advantages and disadvantages of an app, as well as questions relating to emergency notifications regarding severely ill patients’ entries. In addition, we evaluated opinions on data transfer options, data use for scientific purposes, and possible simplification and standardization of follow-up check-ups (see Multimedia Appendix 1: original questionnaire [German]).

One question per page was displayed. Questions were either designed in multiple-choice format with a single answer (forced entry; questions 1, 2, 5, 11, 12, 14, 16-18, 20-23) or multiple answers (forced entry with free-text response option; questions 8-10, 13, 15), as a matrix/rating scale (forced entry; question 6) or free-text mode (optional entry; questions 3, 4, 7, 19, 24). In addition, certain questions were polar questions (questions 2, 5, 12, 14) with branching logic because some queries were related to previous responses. To avoid a central tendency bias, questions in a rating scale mode consisted of an even number of answers. If necessary, technical terms were explained in a footnote. Because all questions were designed with forced entries or optional free text, only completed questionnaires could be submitted by the user and were analyzed. The participant was able to revise answers using a back button.

A sample of 18 experienced professionals in the field of oncology pretested and crosschecked the survey to determine whether the questions were clear and understandable. Consequently, minor changes were made to provide a better understanding and a more user-friendly interface. A link to the survey was sent to HCPs at our hospital via an in-house email distributor representing a convenience sample. The participation was anonymous and voluntary. Approval by the ethics committee and informed consent were not necessary because it was a survey not involving patients.

We conducted the survey for 6 weeks on an online platform (Survio sro, Czech Republic) in March and April 2016 in accordance to the Checklist for Reporting Results of Internet E-Surveys (CHERRIES) guidelines [15]. The platform ensured data protection and security (2048-bit SSL security, ISO/IEC 270001 standards, daily backups). Unique survey visitors were determined by cookies, which were valid depending on particular browser settings. Because the survey was conducted anonymously, we could not prevent users accessing and submitting the survey multiple times.

Statistical calculations were performed using SPSS version 23 (IBM Corp, Armonk, NY, USA) in a primarily descriptive way.

## Results

A total of 108 HCPs (female: n=48; male: n=60) completed the online questionnaire (completion time: median 7.4, range 2.3-322.3 minutes). The survey software counted 290 unique survey visitors, 118 of which only visited the start page and never started the survey and 64 started the survey but did not submit the answers. Hence, the participation rate was 59.1% (172/290) and the completion rate was 37.2% (108/290). Participants’ characteristics are shown in Table 1.

**Table 1:** Participants’ characteristics (N=108).

Characteristic		n (%)
**Gender**	
	Female	48 (44.4)
	Male	60 (55.6)
**Age (years)**	
	20-39	58 (53.7)
	40-59	42 (38.9)
	≥60	8 (7.4)
**Position**	
	Resident	24 (22.2)
	Attending physician	17 (15.7)
	Senior physician	27 (25.0)
	Head of department	8 (7.5)
	Nurse	15 (13.9)
	Other	17 (15.7)
**Medical specialty**	
	Internal medicine	46 (42.6)
	Surgery	42 (38.9)
	Other	20 (18.5)
**Treatment of oncological patients**	
	Yes	83 (76.9)
	No	25 (23.1)
**Scientific background**	
	Yes	88 (81.5)
	No	20 (18.5)

The majority of respondents (88.9%, 96/108) considered telemedicine useful. When asked for advantages of telemedicine, participants named location independence, better documentation of data and test results, improved and continual care for patients in rural areas, enhancement in communication between HCPs and patients, improved patient compliance, the possible use of data for scientific evaluations, and the potential of patient-independent information. In turn, primary disadvantages were concerns about data privacy, loss of the personal visual impression of patients, less time for clinical routine, a possible lack of financial compensation for the service, and the pressure to answer patient requests promptly.

**Figure 1:**
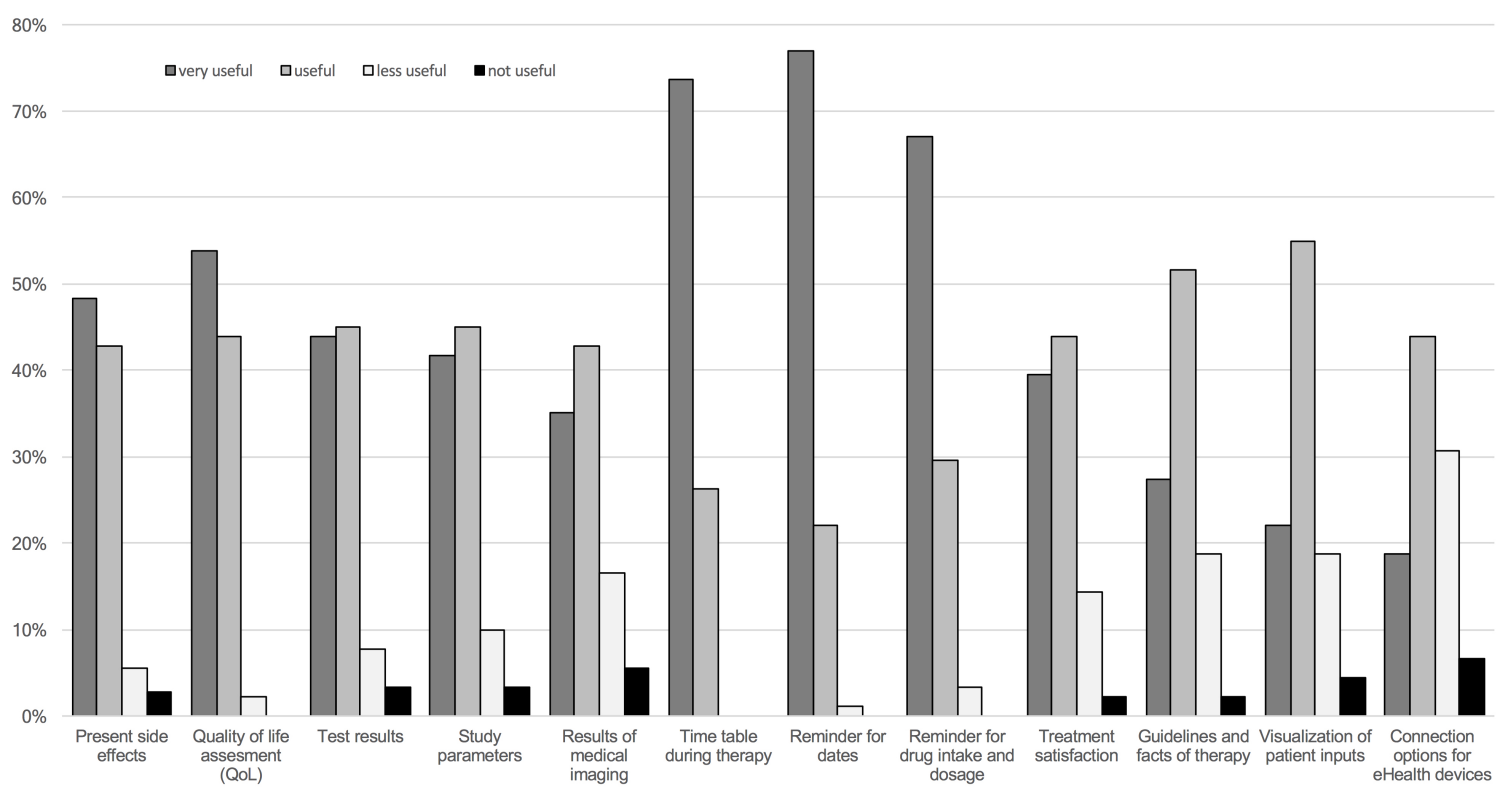
Diagram showing health care providers’ opinion on possible functions for oncological apps (n=91).

In total, 84.3% (91/108) supported the idea of an oncological app complementing classical treatment, whereas 15.7% (17/108) did not regard it as reasonable. If respondents were in favor of oncological apps (n=91), we asked for their opinion on certain functions (Figure 1). Timetables during therapy (eg, dates for chemotherapy or radiotherapy), a reminder for those dates, and a reminder for medication intake and dosage were rated very useful by 74% (67/91), 77% (70/91), and 67% (61/91), respectively, and as useful by 26% (24/91), 22% (20/91), and 30% (27/91), respectively. Assessing quality of life (very useful: 54%, 49/91; useful: 44%, 40/91), current side effects (very useful: 48%, 44/91; useful: 43%, 39/91), and laboratory test results (very useful: 44%, 40/91; useful: 45%, 41/91) were classified as valuable. Further, registering parameters for possible clinical trials (very useful: 42%, 38/91; useful: 45%, 41/91), monitoring treatment satisfaction (very useful: 40%, 36/91; useful: 44%, 40/91), and collecting results of medical imaging (very useful: 35%, 32/91; useful: 43%, 39/91) were also seen as feasible functions. Guidelines and information about current therapy (very useful 28%, 25/91; useful 52%, 47/91) and visuals of patient inputs such as blood results and side effects (very useful: 22%, 20/91; useful: 55%, 50/91) were other functions of high relevance.

**Figure 2:**
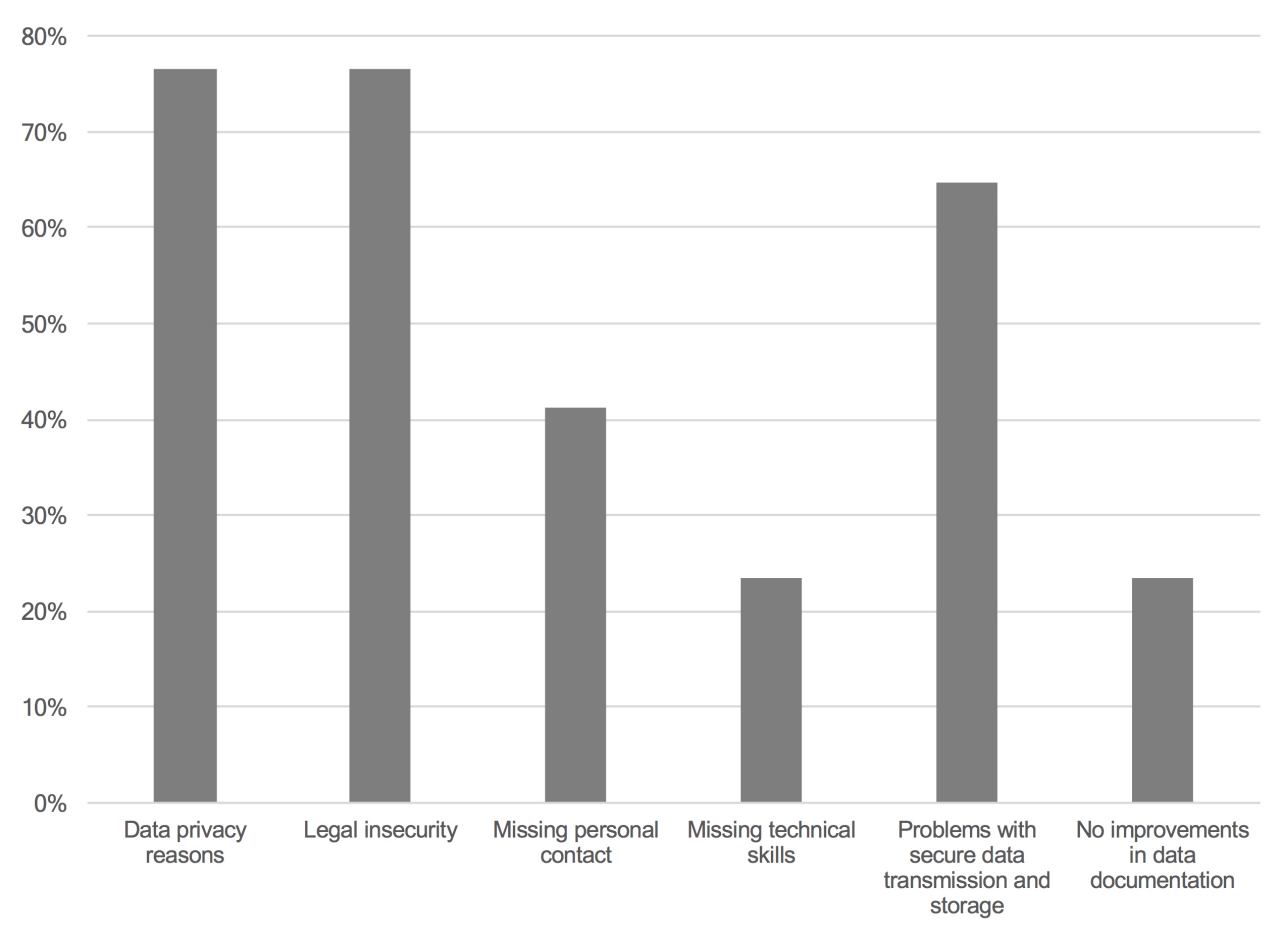
Diagram showing health care providers’ reasons to reject the use of oncological apps (n=17).

All critics not in favor of oncological apps (n=17) specified their motives (Figure 2). As expected, legal uncertainty regarding medical responsibility (77%, 13/17), data privacy issues (77%, 13/17), and possible problems with insecure data transfer and storage (65%, 11/17) were named arguments against establishing an app. The wish for personal contact between HCP and patient (41%, 7/17), missing technical skills (24%, 4/17), and doubt in improvements of data documentation (24%, 4/17) were additional reasons.

Further, we asked the HCPs who considered apps useful (n=91) for their preferred way of data transfer. In all, 75% (68/91) named an encrypted upload to the servers of the clinic as the best possible way, whereas 35% (32/91) preferred a local submission (eg, via offline tablets at the clinic). Cloud storage was favored by 23% (21/91), data transfer via email attachment by 12% (11/91), and 11% (10/91) had no preference. Furthermore, we asked for preferences concerning data export. Direct integration in the hospital information system (74%, 67/91), export for inspection and analysis via PC (59%, 54/91), or mobile device (52%, 47/91) were highly recommended. Paper-based data provision (24%, 22/91) or email (14%, 13/91) were further answers.

Of all respondents, 77.8% (84/108) believed in a clear time savings if the collected data by an app were available for follow-up appointments, whereas 22.2% (24/108) were not convinced of the benefit of app-based patient documentation.

Moreover, we asked questions about an alert function for data inputted by patients requiring an immediate action (eg, severe side effects). Of all, 64.8% (70/108) preferred to be alerted if their patient entered data that needed further clarification, whereas 35.2% (38/108) did not want to be contacted. HCPs in favor of this feature (n=70) were asked for their favorite time interval for making contact. Of these, 49% (34/70) preferred an alarm mechanism for the treating physician within 24 to 48 hours, whereas 40% (28/70) were in favor of an immediate notification of severe cases to the physician on duty, 14% (10/70) preferred an independent query in an implemented alert system, and 27% (19/70) of HCPs chose “no answer.” In addition, most preferred a graded notification from mild to severe. If HCPs were alarmed, 94% (66/70) were willing to contact the patient, whereas 6% (4/70) would refuse to. Reasons were lack of time (3/4), legal insecurity (2/4), and the wish to delegate this task to other staff (1/4).

All respondents were asked about their opinion on using the collected data for scientific evaluations. Of all, 93.5% (101/108) supported it, whereas 6.5% (7/108) did not. Furthermore, we asked all HCPs if they believed an app could be a competitive advantage for the providing hospital. Three-quarters agreed strongly (75.0%, 81/108), whereas 25.0% (27/108) disagreed.

## Discussion

This survey analyzed the attitude of HCPs toward telemedicine, mHealth, and mobile apps in the field of oncology. Using an online questionnaire, we conducted the survey within our oncological center.

Telemedicine is widely accepted in our cohort of HCPs (88.9%, 96/108). Most frequently, participants stated the advantage of being in an independent location and improving the care for patients in rural areas. Especially in Germany, where the health sector faces a shortage of general physicians [16] and a nursing crisis [17] in rural areas, telemedicine could improve the situation. Oncological patients in particular need a close and continual connection to their treating department, as their disease needs accurate observation and, if necessary, a quick response to progression. However, not every town or small city in rural areas offers the same standards of care, and traveling to more developed regions needs time, financial backing, and physical strength. Telemedicine could ease the situation and lower the pressure on highly frequented HCPs in rural areas without decreasing the standard of care. A systematic review of eHealth apps by Banbury et al [18] showed increased access to health care in remote areas, an enhancement in the professional development of HCPs, and lower travel costs. Jhaveri et al [19] evaluated a remote chemotherapy supervision model in a rural area in Queensland, Australia, that enabled rural physicians and nurses to treat patients with telemedical advice from big centers. It showed a better continuity of patient care, reduction of travel costs, shorter waiting times, and importantly no reported adverse events. As telemedicine is based on electronic storage, data can be saved long term and more efficiently and with smaller space compared to paper-based documentation [20].

An improvement of patients’ communication and the possibility to inform themselves about their disease are further important advantages for telemedicine. A higher patient compliance is obtained by a closer link to the treating department and the offered functionality of the app for reminding the patient of things such as follow-up dates, drug intake, or physical exercises. Wang et al [21] designed a randomized controlled trial and showed a higher compliance for patients with esophageal cancer using Internet follow-up after radiotherapy compared to a control group. A 15-year experience with telemedicine in Korea published by Kim et al [22] compared telemedical services for patients versus face-to-face medical service and showed a significant improvement of compliance in drug administration and lifestyle changes. However, HCPs also named certain disadvantages regarding telemedicine. The most mentioned is a possible lack of data protection and violation of privacy. Nowadays, it is possible to encrypt data and transfer it via a highly secure line to a server or cloud [23,24]. Further, the right of medical confidentiality and the right to informational self-determination are not violated by the use of telemedicine. The missed time for clinical routine work and the resulting pressure to answer patient requests promptly concerns many HCPs. As telemedicine offers a wide field of possible features, some of them could even spare time in everyday clinical routine. Nilsson et al [25] sent a nurse to patients who measured their blood pressure and, if necessary, contacted a doctor by video conference. Levy et al [26] asked patients to monitor their blood sugar levels themselves and send the data via text message to a nurse who reviewed them and, if necessary, adjusted the insulin dose. Both evaluations showed at least similar effectiveness as face-to-face contact with doctors. Hence, time-consuming tasks (eg, follow-up appointments) could be replaced and complemented by telemedical services. Further, the pressure for immediate answer via telemedical services can be eased by implementing standardized response times (eg, 24-48 hours in nonsevere cases).

The financial compensation for telemedicine is limited by country laws and local health plan regulations. The current development in Germany points toward increased financial compensation; since 2016, telemedical services in cardiology are billable [27]. A limit of telemedicine is the lack of visual impression of the patient by HCPs. Clinical diagnosis is often based on a long-standing experience and holistic care of the patient. This dimension is missing in telemedical approaches. Hence, initial diagnosis should never be made over such a medium.

In our survey, we investigated mobile apps as a telemedical or mHealth tool for patient-reported symptoms and disease parameters in an oncological setting. Of all participants, 84.3% (91/108) support the idea of an oncological app complementing classical treatment. Assessing side effects present during therapy is one of the most important functions. Giving the patient the opportunity to grade their side effects on a regular basis (eg, weekly) enables the HCP to contact patients in severe cases. Further, the development of side effects over time can be important information, if available at follow-up appointments. Transferring imaging and test findings completes the documentation of the course of the disease and allows for prompt reaction in case of progression. Assessing study parameters (eg, blood pressure, blood sugar, weight) and quality of life is important for scientific evaluations. Gathering treatment satisfaction data improves department workflows and allows for patient-friendly treatment processes.

A timetable and notification system were highly recommended. Reminding patients of chemotherapy or radiotherapy dates could reduce the inconvenience on both sides and improve patient compliance. A reminder of drug intake and dosage could support drug adherence, reduce medication errors, and save time in emergency situations [28,29]. Guidelines and facts during/after therapy (eg, care instructions, diet tips, exercises) could help to improve the treatment process and inform patients about their disease.

Visualization of patients’ input is a feature mostly to improve patients’ compliance because presenting blood results or side effects in graphs gives a better understanding of the course of disease. Further, connection possibilities to other eHealth devices (eg, fitness bands, blood pressure monitor, blood glucose meter) would provide even more detailed information about the patient’s clinical constitution. A possible future functionality could be automated algorithms, which calculate the personal risk profile for disease progression using all the previously named patient inputs. This would be a further step toward holistic, personalized medicine.

Those HCPs not in favor of using an oncological app were mostly afraid of legal uncertainties regarding medical responsibility (77%, 13/17). Each country regulates medical apps and legal responsibility differently. However, the legally required duty of care by HCPs and the right of informed consent by patients should be important values also applied to medical apps.

**Figure 3:**
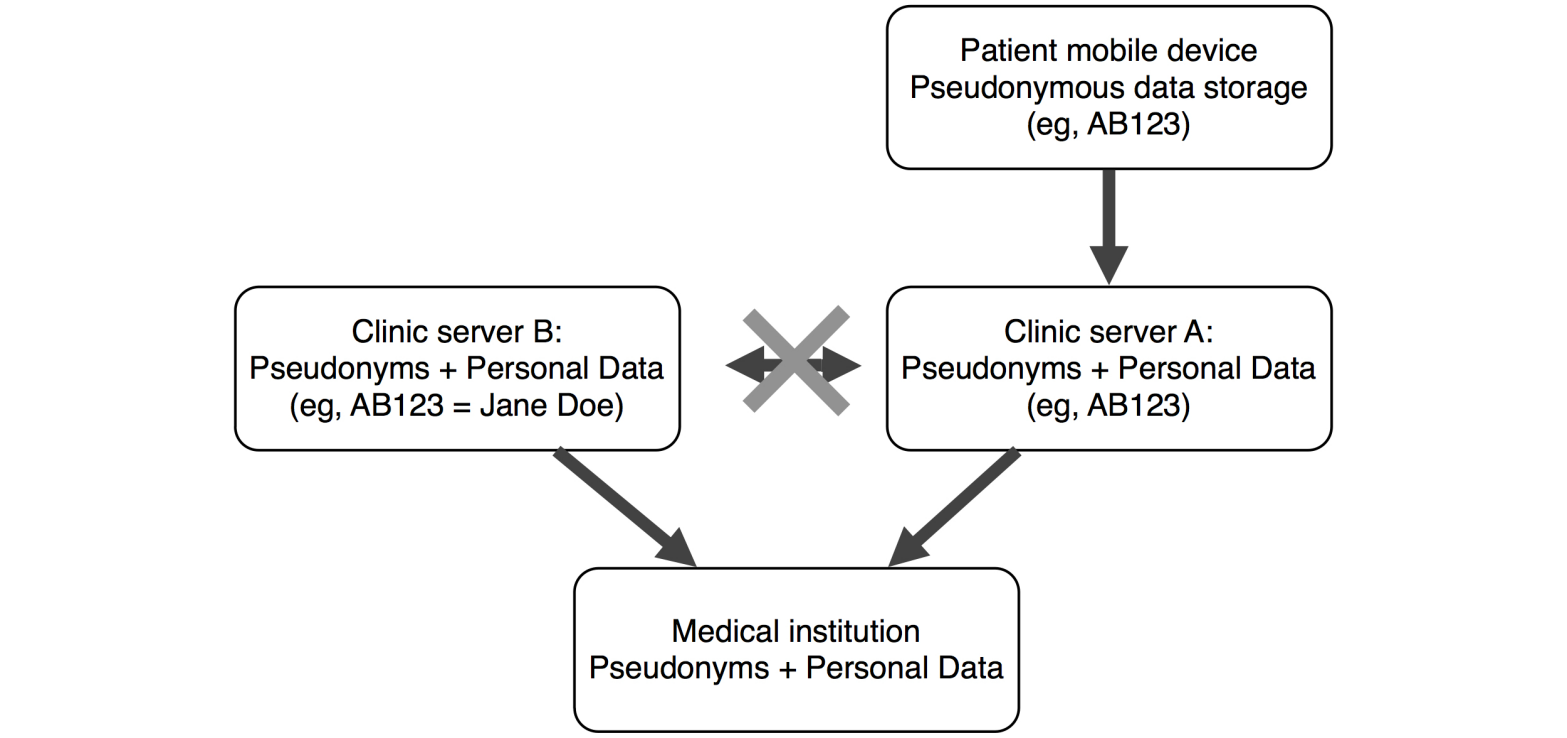
Diagram showing a schematic pseudonymous data transfer to a server at the clinic via a secure line.

Another problem is the fear of data privacy issues (77%, 13/17) and insecure data transfer and storage (65%, 11/17). An anonymous approach is not possible because the medical institution needs to identify the patient. A pseudonymous approach (Figure 3) could be a compromise. Patients receive a pseudonym (eg, AB123) during registration at the clinic. With this pseudonym, the patient logs in to the app and data are stored locally and pseudonymously on the device. Then, pseudonymous data are sent encrypted to a Server A. Only on Server B are stored the pseudonyms in conjunction with personal data of the patient (eg, AB123=Jane Doe), and it is not linked to Server A. Hence, only the medical institution, which has access to Server A and B, can retrieve both pseudonymous and personal data.

A further point of criticism is the missing personal contact between HCP and patient (41%, 7/17). An app can never replace the personal patient-physician relationship, which is an important factor for treatment success. However, an app can reduce unnecessary patient contacts; moreover, it can complement classical treatment.

Of the asked HCPs, 64.8% (70/108) want to be contacted in case of data input that indicates severe and moderate side effects. A possible scenario could be to report those to the physician on duty (40%, 28/70 agree) and treat those immediately. Moderate side effects can be reported to the treating physician (49%, 34/70 agree) within a certain time interval (eg, 24-48 hours) and lead to further treatment or a wait-and-see strategy. Denis et al [11] evaluated high-risk lung cancer patients who filled out weekly Web-based questionnaires. Relapse was detected, on average, 5 weeks before planned restaging. Hence, needed treatment could be started significantly earlier than with standard follow-up procedures. In the case of severe side effects, 94% (66/70) are willing to contact the patient. This would lead to quicker response and earlier treatment of the condition. However, how to define the perfect cut-off between data inputs that indicate severe and moderate side effects remains subject to further investigation.

Another point of interest for HCPs is the scientific value of the collected data. The HCPs (93.5%, 101/108) are keen to evaluate the data and use it for further assessment in diagnostics and the improvement of therapy. Because they choose to perform the survey in a university hospital, the resulting scientific background of the interviewee (81.5%, 88/108 working on scientific projects) might contribute to the high percentage. Furthermore, an app could also be used in prospective trials. Needed clinical visits during long-term randomized controlled trials are always connected with a high organizational workload and depend on the compliance of patients. Certain study parameters could be easily obtained and transferred via a mobile app and could extend the standard retrieved data. The interdisciplinary character would be complemented with a longitudinal approach. Of course, patient compliance and informed consent are important requirements for the success of scientific evaluations. To that, Chen et al [30] showed a general willingness of the public to share data for health research.

This work shows a great approval for telemedicine, mHealth, and apps in oncology among HCPs. Assessing side effects can lead to quicker response and thus lower inconvenience for patients. Clinical data such as life quality and treatment satisfaction could be used to evaluate and improve the therapy workflow. Registered test and medical imaging results can be used to document the disease progression and the collected data can be used for scientific evaluations. Eventually, mobile apps would enhance the patients’ relation to his treating department because they are in permanent contact—a trend also evolving in the medical field.

## Appendix

Multimedia Appendix 1 Original questionnaire (German).

## Abbreviations

CHERRIES: Checklist for Reporting Results of Internet E-Surveys
HCP: health care professional
