# Supplementary material for: Mobile Apps in Oncology: A Survey on Health Care Professionals’ Attitude Toward Telemedicine, mHealth, and Oncological Apps
Source: J Med Internet Res. 2016 Nov 24;18(11):e312. doi: 10.2196/jmir.6399 (PMC5146327; doi:10.2196/jmir.6399)
Supplement: Multimedia Appendix 1 [file jmir_v18i11e312_app1.pdf]

ACHTUNG: Dies ist eine Umfrage Vorschau, eingereichte Antworten werden nicht gespeichert. Klicken Sie hier (https://www.survio.com/survey/d/G6L3V7D9E9S6G7O3P), die Umfrage zu ausfüllen.

Klinikweite Personal-Umfrage zu einer onkologischen App für Smartphones und Tablets

Liebe Kolleginnen, liebe Kollegen,

Smartphone-*Apps* finden immer mehr Anwendung und unterstützen uns im Alltag. Auch in der medizinischen Versorgung kommen vermehrt elektronische / mobile Geräte zum Einsatz – allgemein als *eHealth* bezeichnet.

Eine solche App für Patienten würde viele Möglichkeiten eröffnen. Die gewonnenen Daten können zum Beispiel zur Therapieverbesserung oder wissenschaftlichen Auswertung verwendet werden. Akute Ereignisse (z. B. schwere Nebenwirkungen) könnten zeitnah dokumentiert und dem behandelnden Arzt gemeldet werden. Aber auch die Bereitstellung der Daten zu Nachsorgeuntersuchungen erleichtert die Dokumentation des Therapieverlaufs und verbessert die Nachvollziehbarkeit der Nebenwirkungen und Lebensqualität der Patienten.

Wir möchten gerne herausfinden, wie wir eine App sinnvoll in unserer Klinik zum Vorteil von Patienten und Personal einsetzen könnten. Hierfür benötigen wir Ihre Hilfe und Meinung! Wir würden uns daher freuen, wenn Sie sich **10 Minuten** Zeit nehmen, um an unserer Umfrage zu einer Gesundheits-*App* für Patienten in der Onkologie für Smartphones und Tablets teilzunehmen. So können Sie uns bei der Gestaltung der App unterstützen. Eine ähnliche Umfrage wird gerade bei onkologischen Patienten durchgeführt, die im Klinikum rechts der Isar behandelt werden.

Alle Ihre Angaben sind anonym und werden natürlich streng vertraulich behandelt.

Wir danken Ihnen sehr für Ihre Mithilfe!

Mit freundlichen kollegialen Grüßen

Univ.-Prof. Dr. med. Stephanie E. Combs

Leiterin des Onkologischen Zentrums, RHCCC

1

Behandeln Sie onkologische Patienten?

- ◯ Ja
- ◯ Nein

2

Finden Sie Telemedizin als Ergänzung zum regulären Patientenkontakt sinnvoll?

Die Telemedizin bezeichnet Diagnostik, Therapie und Nachsorge unter Überbrückung einer räumlichen oder auch zeitlichen Distanz zwischen Arzt (Telearzt) und Patienten.

- ◯ Ja
- ◯ Nein

3

Was spricht Ihrer Meinung nach insbesondere für Telemedizin? (optionale Angabe)

4

Was spricht Ihrer Meinung nach gegen Telemedizin? (optionale Angabe)

5

Würden Sie die Nutzung einer onkologischen App durch Patienten begleitend zur Therapie befürworten?

- ◯ Ja
- ◯ Nein

6

Wenn ja, wie würden Sie folgende Funktionen einer onkologischen App für Patienten bewerten?

	nicht sinnvoll	weniger sinnvoll	sinnvoll	sehr sinnvoll
regelmäßige Abfrage aktueller Nebenwirkungen	◯	◯	◯	◯
Dokumentation der Lebensqualität (QoL)	◯	◯	◯	◯
Laborergebnisse (insb. externe)	◯	◯	◯	◯
Studienparameter (z.B. Gewicht, Blutzuckerwerte, Blutwerte, Blutdruck, Puls, Temperatur...)	◯	◯	◯	◯
Befunde zur Bildgebung (insb. externe)	◯	◯	◯	◯
Terminkalender während Therapie (z. B. für Chemotherapie- oder Bestrahlungstermine)	◯	◯	◯	◯
Erinnerung an Termine (Bestrahlungstermine, Nachsorgetermine, etc.)	◯	◯	◯	◯
Erinnerung an Medikamenteneinnahme und Dosierungen	◯	◯	◯	◯
Therapiezufriedenheit	◯	◯	◯	◯
Nachschlagewerk und Wissenswertes zur Therapie (Pflegehinweise, Übungen, Wegepläne, etc.)	◯	◯	◯	◯
Visualisierung der Eingaben (Laborwerte als Tabelle, Verlauf der Nebenwirkungen, etc.)	◯	◯	◯	◯
Verbindungsmöglichkeit von eHealth-	◯	◯	◯	◯

Geräten (z. B. Fitnessarmbänder, Blutdruckmessgeräten, Zuckermessgeräte, Waage)

7

Fallen Ihnen noch weitere Funktionen ein? (Angaben optional)

8

Wenn nein, was halten Sie für die größten Schwierigkeiten bzw. Gründe die gegen eine App sprechen?

- [ ] Datenschutzgründe
- [ ] rechtliche Verantwortlichkeit/Verfügbarkeit bei akuten Beschwerden/Meldungen des Patienten
- [ ] Ich will nur persönlichen Kontakt zu meinen Patienten.
- [ ] Ich kenne mich mit der Technik nicht aus.
- [ ] Problem der sicheren Datenübertragung und -speicherung
- [ ] Wird die Dokumentation zum Krankheitsverlauf nicht verbessern
- [ ] Andere

9

Welche Art der Übermittlung der Patientendaten mit nachträglicher Bereitstellung in einem System würden Sie bevorzugen?

Unter Cloud Speicherung versteht man das Speichern von Daten in einem entfernten Rechenzentrum, also nicht auf dem lokalen Arbeitsplatzcomputer oder Server, sondern entfernt in der (metaphorischen) Wolke (englisch: cloud).

- [ ] direkt an einen Server in der Klinik (in verschlüsselter Form)
- [ ] per E-Mail Anhang
- [ ] Speicherung in einer Cloud
- [ ] Lokale Datenübertragung: Eingabe direkt vor Ort z. B. per Tablet in der Klinik
- [ ] egal
- [ ] Andere

10

Welche Benutzeroberfläche zur ärtzlichen/pflegerischen Abfrage der Daten würden Sie sich wünschen?

- [ ] Ausgabe auf mobilen Endgeräten
- [ ] Ausgabe auf dem PC
- [ ] Einbindung/Ausgabe im SAP-Kliniksystem
- [ ] Ausgabe als E-Mail Anhang
- [ ] Ausgabe in Papierform in der Patientenakte
- [ ] Andere

11

Könnte die Bereitstellung von Patientendaten durch eine App Ihrer Meinung nach zu einer Zeitersparnis bei Nachsorgeuntersuchungen führen?

- ○ Ja
- ○ Nein

12

In Bezug auf die Dokumentation von Nebenwirkungen durch den Patienten selbst: Würden Sie ein Benachrichtigungs-System zur (sofortigen) Benachrichtigung befürworten?

- ○ Ja
- ○ Nein

13

Wenn ja, in welchem Zeitraum soll die Benachrichtigung erfolgen?

- [ ] sofort in der Dienstzeit an den Dienstarzt (bei akuten Fällen)
- [ ] innerhalb von 24h - 48h an einen betreuenden Arzt
- [ ] eigenständige aktive Abfrage (in einem System)
- [ ] Andere

14

Wären Sie bereit Patienten aufgrund einer Benachrichtigung zu kontaktieren?

- ○ Ja
- ○ Nein

15

Wenn nein, warum sind sie gegen eine Kontaktaufnahme?

- [ ] Für so etwas habe ich keine Zeit.
- [ ] Diese Aufgabe soll anderes Personal erledigen.
- [ ] Ich bin mir nicht über die rechtlichen Folgen einer solchen Kontaktaufnahme bewusst.
- [ ] Andere

16

Sind Sie in der Wissenschaft tätig oder betreuen Sie Studien?

- ○ Ja
- ○ Nein

17

Würden Sie es sinnvoll finden, wenn die gesammelten Daten zur wissenschaftlichen Auswertung (u.a. zur Studiendokumentation) verwendet werden könnten?

- ○ Ja
- ○ Nein

18

Glauben Sie, die App würde einen Wettbewerbsvorteil gegenüber anderen Kliniken bieten?

- ○ Ja
- ○ Nein

19

Haben Sie einen Namensvorschlag für die App?

20

Welches Geschlecht haben Sie?

Auswählen

21

Wie alt sind Sie?

- <20
- 20-29
- 30-39
- 40-49
- 50-59
- >=60

22

In welcher Position sind Sie tätig?

Auswählen

23

In welcher Abteilung des Klinikum rechts der Isar sind Sie beschäftigt?

Auswählen

24

Haben Sie weitere Vorschläge für die App oder Kommentare zur Umfrage?

ABSENDEN

Kostenlos **Umfrage erstellen** (http://www.survio.com/de/?source=survey_footer&medium=link&term=survey_link) ✓ Powered by Survio (http://www.survio.com/de/merkmale-funktionen?source=survey_footer&medium=link&term=brand)
